# Aggregation-Enhanced Photophysical Performance of D-π-A Structured Hemicyanine for NIR-II Fluorescent and Photoacoustic Imaging-Guided Photothermal Therapy

**DOI:** 10.1021/cbmi.2c00004

**Published:** 2023-03-06

**Authors:** Baoling Li, E Pang, Shaojing Zhao, Guowei Deng, Shuodong Wang, Benhua Wang, Jieyun Wu, Guangle Niu, Xiangzhi Song, Minhuan Lan

**Affiliations:** † Hunan Provincial Key Laboratory of Micro & Nano Materials Interface Science, College of Chemistry and Chemical Engineering, Central South University, Changsha, Hunan 410083, P. R. China; ‡ College of Chemistry and Life Science, Sichuan Provincial Key Laboratory for Structural Optimization and Application of Functional Molecules, Chengdu Normal University, Chengdu, Sichuan 611130, P. R. China; § State Key Laboratory of Chemo/Biosensing and Chemometrics, College of Chemistry and Chemical Engineering, Hunan University, Changsha, Hunan 410082, P. R. China; ∥ School of Optoelectronic Science and Engineering, University of Electronic Science and Technology of China, Chengdu, Sichuan 611731, P. R. China; ⊥ State Key Laboratory of Crystal Materials, Shandong University, Jinan, Shandong 250100, P. R. China

**Keywords:** NIR-II fluorescence, donor-π-acceptor structure, hemicyanine, aggregation, photothermal therapy

## Abstract

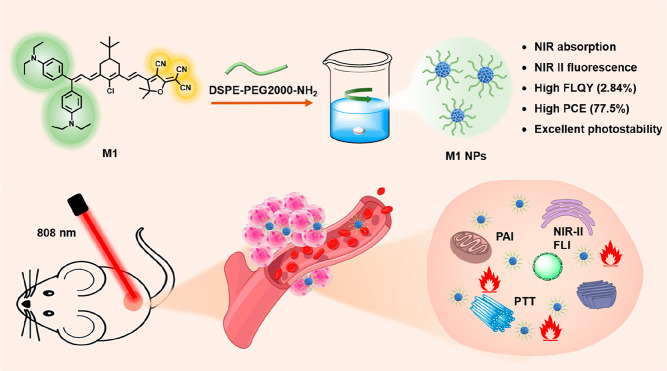

Near-infrared (NIR)-II fluorescence and photoacoustic (PA) dual-model imaging-guided photothermal therapy (PTT) can precisely diagnose and treat tumors and evaluate the therapeutic efficacy in real-time. Herein, we utilized a donor-π-acceptor (D-π-A) structured hemicyanine dye (named M1) with a large conjugated structure and strong intramolecular charge transfer effect and demonstrated that the aggregation of M1 could significantly enhance its photophysical performance by improving its photostability and photothermal conversion capability as compared with M1 in a single molecular state. Furthermore, we prepared water-dispersible NIR-II fluorescent nanoparticles (M1 NPs) by wrapping M1 with DSPE-PEG2000-NH_2_. The obtained M1 NPs exhibit strong NIR-I absorption and NIR-II fluorescence with their maxima at 734 and 1040 nm, respectively, with a fluorescence quantum yield of 2.84%. Moreover, M1 NPs also exhibit excellent biocompatibility, good photostability, and high photothermal conversion efficiency of 77.5%. *In vitro* and *in vivo* experiments reveal that M1 NPs can effectively image tumors through NIR-II fluorescence and PA signals, inhibit DNA replication, trigger cytoskeleton collapse, and eventually induce tumor cell apoptosis under 808 nm laser irradiation. Based on these outstanding properties, the application of M1 NPs in PA and NIR-II fluorescence imaging-guided PTT of tumors is demonstrated.

## 1 Introduction

High-quality imaging holds great promise for cancer diagnosis and treatment.^1,2^ Near-infrared (NIR)-II fluorescence imaging, an optical imaging modality, is capable of high image resolution and real-time monitoring but suffers from low tissue penetration ability.^3,4^ In contrast, photoacoustic (PA) imaging technology uses the ultrasonic wave as the carrier to obtain the optical absorption information on tissues and replaces photon detection in traditional optical imaging with ultrasonic detection, thus avoiding the problem of insufficient penetration depth caused by optical scattering, breaking through the soft limit of traditional optical imaging (about 1 mm), and realizing PA imaging of deep tissues with a depth of up to 7 cm.^5−7^ However, the imaging resolution of PA is lower than fluorescence. Therefore, imaging techniques that combine NIR-II fluorescence and PA imaging afford higher-quality photographs than a single technique.^8,9^

Photothermal therapy (PTT) has attracted widespread attention due to its high selectivity, non-invasiveness, and oxygen-independent features.^10,11^ A precise diagnosis and therapy of tumors can be achieved simultaneously by NIR-II fluorescence and PA imaging-guided PTT, along with the evaluation of the therapeutic efficacy in real-time. Ideal phototherapeutics are characterized by strong NIR absorption/fluorescence and high photothermal conversion efficiency (PCE).^12,13^ Metal-based nanostructures usually exhibit high PCE in the NIR region due to their strong plasma resonance. However, the apparent cytotoxicity and absence of fluorescence limit their clinical application.^14^ Although carbon nanostructures exhibit better biocompatibility than metal-based nanostructures, they are non-biodegradable with poor absorption and fluorescence in the NIR region.^15,16^ In contrast, organic phototheranostics, such as coumarin, fluorescein, naphthalimides, rhodamine, and cyanine derivatives, are widely used in fluorescent sensing, bioimaging, and medical therapeutic applications due to their good biodegradability, modifiable molecular structure, and tunable optical properties.^17^ One of the organic phototheranostics, indocyanine green (ICG), has been approved by the FDA for clinical imaging and phototherapy of cancer.^18^ However, this acceptor-π-acceptor structured molecule suffers from poor photostability, low PCE and fluorescence quantum yield, and short absorption and fluorescence wavelengths.

In contrast, molecules with a donor-π-acceptor (D-π-A) structure contain electron-donating and electron-withdrawing groups, as well as large π-conjugated structures, endowing them with strong intramolecular charge transfer (ICT) effect, which red-shifts their absorption and fluorescence.^19^ Hemicyanine dyes, which feature a D-π-A motif, are extensively applied in fluorescence imaging and biolabeling because of the advantages of high molar absorption coefficient, large Stokes shift, good biocompatibility, easy molecular modification, and adjustable absorption and fluorescence wavelengths.^20^ Therefore, by tuning the electron donor and acceptor groups or enlarging the π-conjugated structure, NIR-fluorescent derivatives of hemicyanine could be designed and synthesized.^21^ However, developing novel hemicyanine dyes with strong NIR-II fluorescence and high PCE is still desirable.

**Scheme 1:**
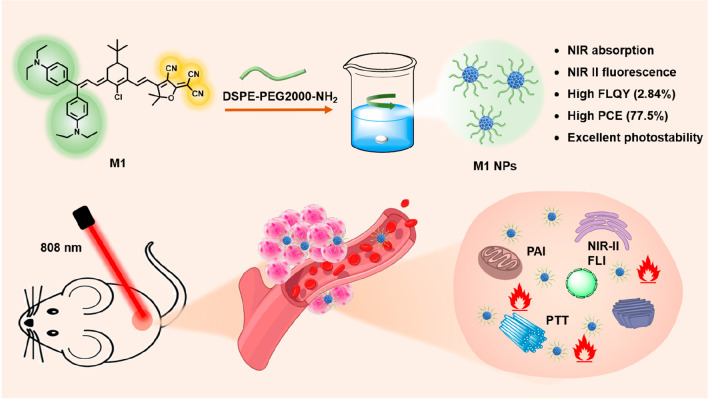
Schematic Preparation and Application of M1 NPs

Based on the above considerations, we selected a D-π-A structured hemicyanine dye, named M1 which has been reported in our previous paper,^22^ as shown in Scheme 1. The molecular design strategy featured in this work has the following advantages: (i) M1 contains three cyano groups as the electron receptors, two diethylamino groups as the electron donors, and six C=C bonds as the π-conjugated system. This large D-π-A structured M1 has a strong ICT effect, exhibits strong NIR absorption and fluorescence, and has high PCE due to the energy gap law.^23^ (ii) The strong electron-withdrawing groups, including three cyano and one chloro, could effectively decrease the electron density in the conjugated structure, thus reducing the photooxidation activity and significantly improving the photostability of M1. The cyclohexene and furan groups also enhance the rigidity of the molecular structure and further improve the photostability of M1. (iii) The *tert*-butyl group on cyclohexene and the two methyl groups on furan can effectively prevent the π–π stacking of M1, improving its fluorescence quantum yield. (iv) The two *N*,*N*-diethylaminophenyl groups are free to rotate and thus improve the non-radiative conversion and increase the PCE of M1.

Moreover, the aggregation of M1 in aqueous solution results in better photostability and higher photothermal conversion capability, as compared with M1 in DMSO. The aggregated M1 could isolate O_2_ to reduce oxidation of the excited state, leading to enhanced photostability than single molecules. On the other hand, the enhanced photostability together with the strong intermolecular interactions of the aggregated M1 also promoted the photothermal conversion capabilities. Hence, water-dispersible NIR-II fluorescent nanoparticles (M1 NPs) were prepared by utilizing the aggregation-enhanced photophysical performance of M1 by wrapping the hydrophobic M1 with DSPE-PEG2000-NH_2_. M1 NPs exhibit strong NIR-I absorption at 734 nm and NIR-II fluorescence at 1040 nm, with a large Stokes shift of 306 nm. The fluorescence quantum yield of M1 NPs in an aqueous solution is measured to be 2.84%, much higher than the previously reported NIR-II fluorescent chromophores,^24^ providing them with excellent NIR-II fluorescence imaging capability. Besides, M1 NPs exhibit excellent biocompatibility, good photostability, and a high PCE of 77.5%, which are superior to the clinically used agent ICG. *In vitro* results revealed that the heat generated from M1 NPs exposed to laser irradiation could effectively inhibit the replication of tumor cell DNA and trigger the collapse of the tumor cytoskeleton, thus inducing apoptosis. Based on their excellent PA and NIR-II fluorescence imaging capabilities, high PCE, good biocompatibility, and photostability, M1 NPs were applied to PA and NIR-II fluorescence imaging-guided PTT.

## 2 Experimental Section

### 2.1 Materials and Instrutments

DSPE-PEG2000-NH_2_ was acquired from Hunan Huateng Pharmaceutical Co. Ltd. 9,10-Anthracenediyl-bis(methylene) dimalonic acid (ABDA) was purchased from Shanghai Aladdin Biochemical Technology Co., Ltd. Dihydrorhodamine 123 (DHR123) was purchased from Dalian Meilun Biotech Co., Ltd. Terephthalic acid (TA) was purchased from Shanghai McLean Biochemical Technology Co., Ltd. MTT, calcein-AM, and propidium iodide (PI) were purchased from Heowns. FITC-labeled phalloidin was ordered from MesGen Biotechnology Co. Ltd. Hoechst 33342 and azide 488 were obtained from Thermo Fisher Scientific. Annexin V-FITC/7-AAD was obtained from Procell Co. Ltd.

The UV–vis–NIR absorption and fluorescence spectra were recorded on Shimadzu UV2600 and RF6000 spectrophotometers, respectively. The fluorescence quantum yield of M1 NPs was measured from full-function fluorescence spectrometer (FLS1000). Scanning electron microscope (SEM) images were obtained from JSM-7610F, JEOL Ltd. Fluorescence imaging of the cells was performed under a confocal laser scanning microscope (Leica SP8) or an inverted fluorescence microscope (Leica DMIL LED).

### 2.2 Preparation of M1 NPs

The detailed synthesis and characterization of M1 are given in our previous report.^22^ Briefly, a mixture of DSPE-PEG2000-NH_2_ (5 mg) and M1 (1 mg) dissolved in THF (1 mL) was injected into deionized water (8 mL) under ultrasound. After removing THF by the rotary evaporator, M1 NPs were obtained by filtering through the 0.22 μm polyether sulfone filter membrane. The concentration of M1 NPs was calibrated from Figure S1.

### 2.3 Reactive Oxygen Species Detection

For singlet oxygen detection, 1.0 mg of ABDA was accurately weighed and dissolved in sodium hydroxide (2 mg/mL, 1 mL), 10 μL of ABDA sodium salt was added to M1 NPs (10 μM) or water. The UV–vis absorption spectra of ABDA sodium salt aqueous solution in the absence and presence of M1 NPs were recorded every 2 min under 808 nm laser irradiation (1 W/cm^2^).

For hydroxyl radical detection, 1.0 mg of TA was accurately weighed and dissolved in sodium hydroxide (2 mg/mL, 1 mL). 10 μL of TA sodium salt was added to M1 NPs (10 μM) or water. The fluorescence spectra of TA sodium salt aqueous solution in the absence and presence of M1 NPs were recorded every 2 min under 808 nm laser irradiation (λ_ex_ = 320 nm, 1 W/cm^2^).

For superoxide anion detection, 5 μL of DHR123 was dissolved in M1 NPs (10 μM) or water. The fluorescence spectra of DHR123 aqueous solution in the absence and presence of M1 NPs were recorded every 2 min under 808 nm laser irradiation (λ_ex_ = 500 nm, 1 W/cm^2^).

### 2.4 Cytotoxicity and Phototoxicity of M1 NPs

Different concentrations of M1 NPs were incubated with 4T1 cells for 24 h, and then the standard MTT assay was used to evaluate the cytotoxicity of M1 NPs. The phototoxicity of M1 NPs was studied by incubating 4T1 cells with M1 NPs for 4 h and subsequent exposure to 808 nm laser irradiation for 10 min with the laser power of 1 W/cm^2^. Then standard MTT assay was performed.

### 2.5 Calcein-AM/PI Staining

4T1 cells were inoculated into 96-well plates for 24 h and divided into four groups based on their subsequent treatment: PBS, PBS + laser, M1 NPs, and M1 NPs + laser (10 μM, 200 μL of M1 NPs or 200 μL of PBS). After 4 h, the cells in laser groups were exposed to an 808 nm laser (1 W/cm^2^) irradiation for 5 min and then incubated for another 30 min. The cells were then washed with PBS, and calcein-AM and PI were added for incubation for 10 min. The cells were finally washed three times with PBS and then imaged under an inverted fluorescence microscope.

### 2.6 FITC-Labeled Phalloidin Experiment

4T1 cells were divided into 4 groups as described above and inoculated into confocal dishes for 24 h. Each group underwent its respective treatment: PBS, PBS + laser, M1 NPs, and M1 NPs + laser (10 μM, 200 μL of M1 NPs or 200 μL of PBS). After 4 h, the cells in laser groups were irradiated for 5 min with an 808 nm laser (1 W/cm^2^). Then 200 μL of FITC-labeled phalloidin solution was added to each group and incubated for 30 min. Finally, the cells were washed three times with PBS and then imaged under a laser confocal scanning microscope.

### 2.7 EdU Staining of Proliferating Cells

4T1 cells divided into 4 groups described above were inoculated into confocal dishes for 24 h, and each group was treated accordingly. 4 h later, the cells in laser groups were exposed to an 808 nm laser (1 W/cm^2^) for 5 min. The cells were then incubated with a mixture of 10 μM EdU and culture medium for 1 h. After incubation was complete, the medium was removed and 50 μL of 4% paraformaldehyde fixative was added and incubated for 15 min. The fixative was then removed after incubation was complete. Next, 50 μL of glycine (2 mg/mL) was added to each group of cells and incubated for 5 min to neutralize any residual fixative. Then 100 μL of 0.5% Triton X-100 was added and incubated for 20 min.

For preparing Click-iT working solution, 860 μL of Click-iT EdU reaction buffer, 40 μL of copper sulfate and 2 μL of Azide-488 were mixed. 100 μL of Click-iT working solution was added to each well and incubated for 30 min at room temperature.^25^ The cells were washed twice with PBS before adding 100 μL of Hoechst 33342 solution and subsequent incubation for 30 min. Finally, cells were washed three times with PBS and imaged with a confocal scanning microscope.

### 2.8 Construction of Tumor-Bearing Mouse Model

All animal experiments were performed under the permission of the Ethics Committee of Central South University (No. 430727211101478756). Balb/c male mice (7–8 weeks) were obtained from SJA Laboratory Animal Co. Ltd. The 4T1 cells were inoculated in 8 boxes of 90 mm culture dishes. After the 4T1 cells grew to 90% of the culture dish, the cells were digested, centrifuged, and then diluted into 2 mL of culture medium. Two mice were selected to be subcutaneously injected with a mixture of 4T1 cells and culture medium (1 mL/each) on their backs to establish a 4T1 tumor-bearing mouse model. After the tumors grew to 800 mm^3^ in about 15 days, the 4T1 tumors were peeled off and cut into 2 mm × 2 mm sized small tumor pieces. These tumor pieces were implanted in mice, and after 10 days later, the tumor grew to ∼100 mm^3^.

### 2.9 *In Vivo* Fluorescence Imaging and PA Imaging

The fluorescence and PA imaging pictures were obtained from two tumor-bearing mice. The mice were intratumorally injected of 100 μL of M1 NPs (10 μM), and after 30 min later, the fluorescence image was obtained from a multifunctional imaging analysis system (Perkin Elmer, IVIS Lumina III), while the PA image was acquired from a photoacoustic computerized tomography scanner (inVision256-TF).

### 2.10 *In Vivo* PTT of Tumors

Twenty 4T1 tumor-bearing mice were randomly divided into four groups (5 mice in each group), named saline, saline + laser, M1 NPs, and M1 NPs + laser. Saline (200 μL) or M1 NPs aqueous solution (10 μM, 200 μL) was injected intratumorally into the mice. The saline + laser group and M1 NPs + laser group were irradiated with 808 nm laser (1 W/cm^2^) for 10 min. The temperature of the tumor under laser irradiation was recorded simultaneously by infrared thermography. The tumor volume and body weight of the mice were measured every 2 days for 14 days. At the end of treatment, the tumors were peeled off for tissue analysis.

### 2.11 Tissue Section Experiments

Four 4T1 tumor-bearing mice were treated with saline, saline + laser, M1 NPs, M1 NPs + laser (7 μM, 200 μL of M1 NPs or 200 μL of saline) and then immediately peeled of tumor tissue and fixed with 4% paraformaldehyde. Next H&E and Ki67 stained sections were performed at Wuhan Sevier Biotechnology Co. Ltd.

## 3 Results and Discussion

**Figure 1:**
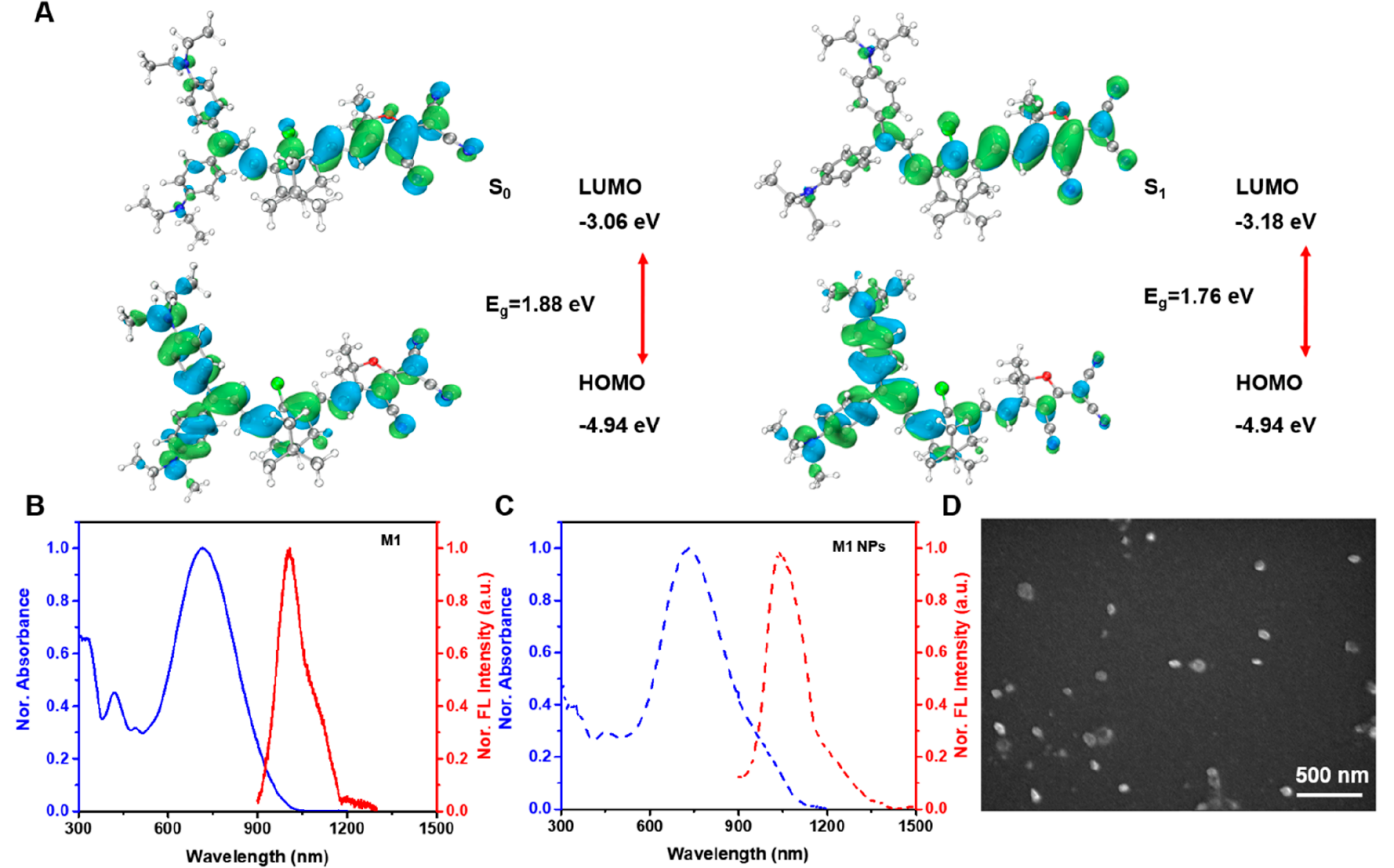
(A) HOMO–LUMO distribution of the ground (left) and excited (right) states of M1. Normalized absorption and fluorescence spectra of (B) M1 in THF and (C) M1 NPs in aqueous solution. (D) SEM image of M1 NPs.

The molecular structure of M1 is shown in Scheme 1. The spatial electron cloud distribution of M1 was analyzed using density functional theory (DFT). As illustrated in Figure 1A, the electrons of HOMOs show very similar distribution, mainly on the diethylamino-phenyl units both in the ground state (left) and the excited state (right). Contrarily, the electrons of LUMOs are apparently delocalized on the cyano-substituted areas, indicating strong ICT. Notably, the molecular configuration, especially the diethylamino portions, shows distinct changes from the ground to the excited state, inducing a low energy gap (*E*_g_) and a large Stokes shift in M1. The calculated *E*_g_ of the ground and excited states is 1.88 and 1.76 eV, respectively.^26,27^

M1 exhibits absorption and fluorescence maxima at 714 and 1005 nm, respectively, in THF (Figure 1B). After encapsulating with DSPE-PEG2000-NH_2_, the obtained water-dispersible M1 NPs show a red shift in absorption and fluorescence spectra, as evidenced in Figure 1C, with peaks at 734 and 1040 nm, respectively. The large Stokes shift of 306 nm is probably due to the strong intermolecular D–A interactions in the aggregate states, further narrowing the *E*_g_ of M1 NPs. The fluorescence quantum yield of M1 NPs in an aqueous solution is measured to be 2.84%. The scanning electron microscope (SEM) image shown in Figure 1D indicates that the M1 NPs have a uniform spherical shape with a mean size of 100 nm, which facilitates cellular uptake. Besides, dynamic light scattering data within 7 days demonstrated excellent stability of the M1 NPs (Figure S2).

**Figure 2:**
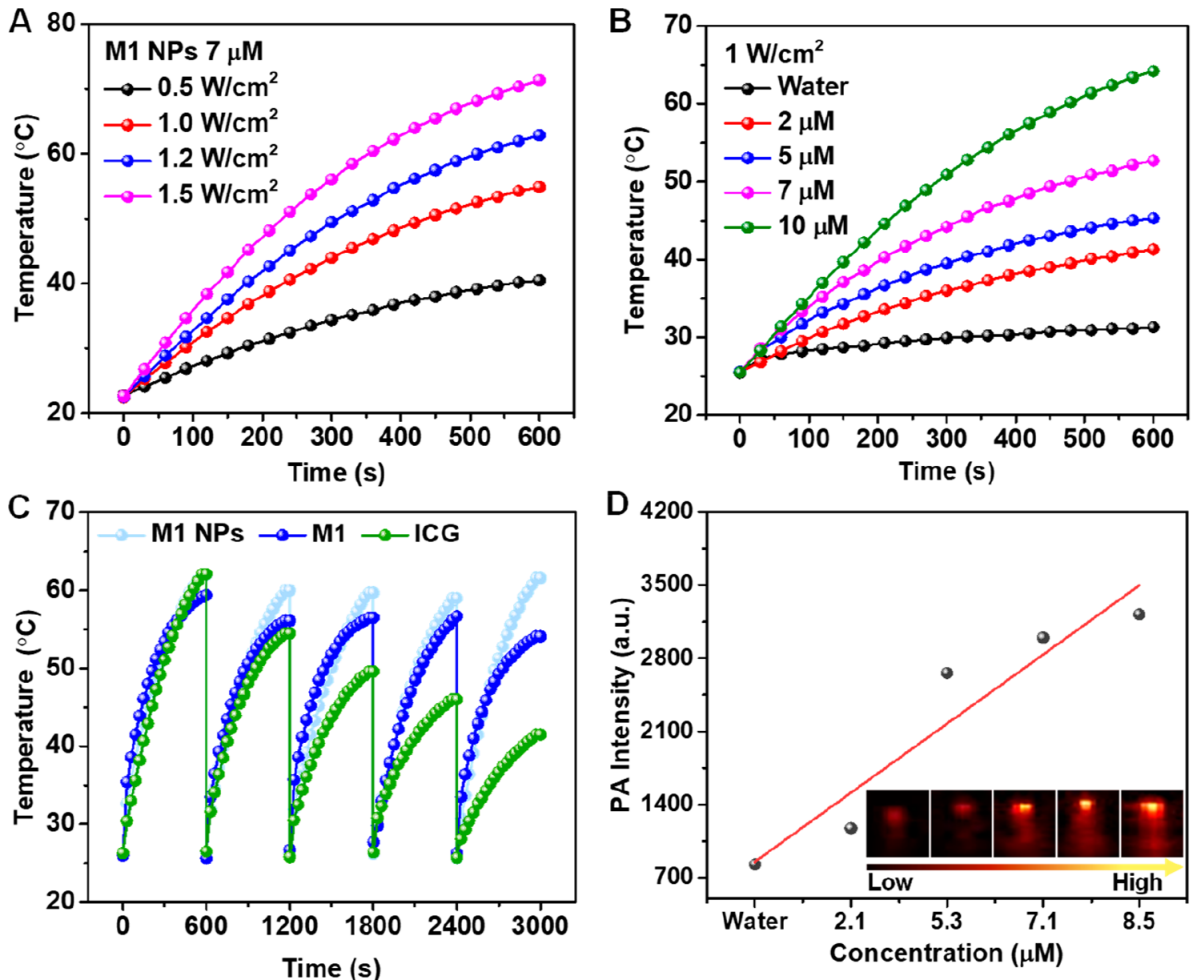
Time-dependent temperature variation of M1 NPs in aqueou solution under laser irradiation at different (A) laser power and (B) concentration of M1 NPs. (C) Temperature variation of M1 NPs in aqueous solution, M1 in DMSO, and ICG in aqueous solution under 808 nm laser irradiation (1 W/cm^2^) for five laser on/off cycles. (D) M1 NP concentration-dependent PA intensity.

Photothermal conversion capability is an important parameter for phototheranostics. As observable in Figure 2A,B, the temperature of M1 NP aqueous solution depends upon laser power and M1 NP concentration. Elevating the laser power or M1 NP concentration increases the temperature significantly. The temperature of the M1 NP solution (10 μM) was increased to 64 °C under 808 nm laser irradiation for 10 min with the laser power of 1.0 W/cm^2^. The infrared thermography results of the M1 NP aqueous solutions of different concentrations after 808 nm laser irradiation for 10 min, shown in Figure S3, are consistent with the above results. The calculated PCE of the M1 NPs is 77.5% (Figure S4),^28^ which is comparable to that of metal-based photothermal agents.^29^ Furthermore, we also investigate the capability of M1 NPs to generate the reactive oxygen species (ROS) under laser irradiation. ABDA sodium salt, TA sodium salt, and DHR123 were used as trappers for the detection of singlet oxygen (^1^O_2_), hydroxyl radicals (^●^OH), and superoxide anions (O_2_^–●^). As shown in Figure S5, the UV–vis absorption spectra of ABDA sodium salt aqueous solution, the fluorescence spectra of TA sodium salt and DHR123 aqueous solutions showed no significant changes in the absence and presence of M1 NPs, suggesting M1 NPs cannot generate ^1^O_2_, ^●^OH, and O_2_^–●^ under 808 nm laser irradiation.

Interestingly, M1 NPs in an aqueous solution exhibit better photostability and photothermal conversion capability than M1 in DMSO. As evident in Figure 2C, after five cycles of laser irradiation, the temperature of the aqueous solution of M1 NPs remained above 60 °C, while the temperature of the DMSO solution of M1 was less than 60 °C. The UV–vis–NIR absorption spectrum of an aqueous solution of M1 NPs showed no obvious changes upon laser irradiation, while that of M1 in DMSO showed a slight decrease in absorbance (Figure S6). Moreover, M1 NPs also have a photostability better than that of ICG, as revealed in Figure 2C and Figure S6. Due to their high PCE in the NIR region and excellent photostability, M1 NPs exhibit PA imaging capability. Furthermore, a strong correlation was found between PA intensity and the concentration of M1 NPs aqueous solution, as observed in Figure 2D.

**Figure 3:**
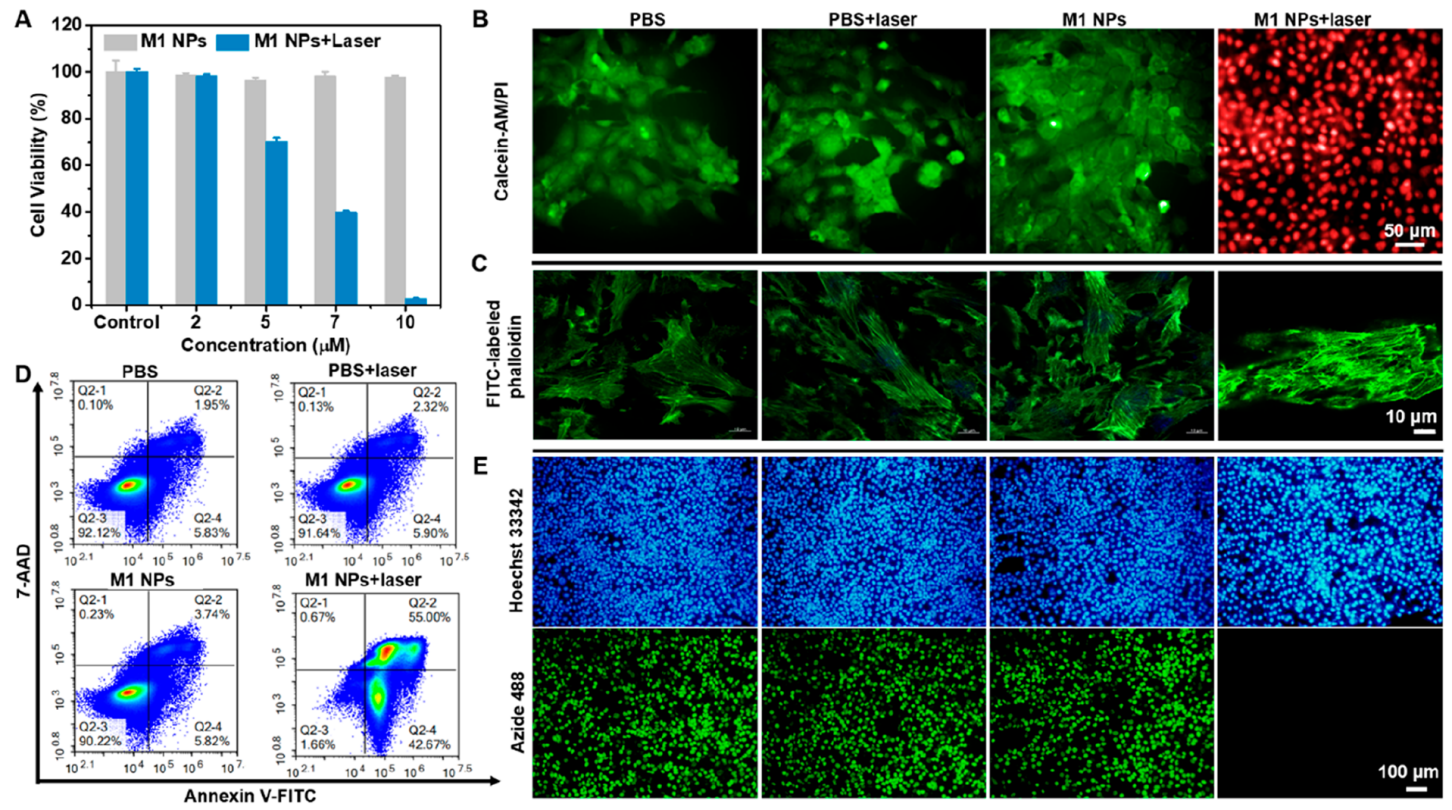
(A) Concentration-dependent cytotoxicity and phototoxicity of M1 NPs. Fluorescence imaging of 4T1 cells after different treatments and staining with (B) calcein-AM/PI and (C) FITC-labeled phalloidin. (D) Flow cytometry and (E) fluorescence imaging of EdU staining of 4T1 cells after different treatments.

The cytotoxicity and phototoxicity of M1 NPs were studied via MTT assay. The viability of 4T1 cells remained above 95% in the absence of laser irradiation even when the concentration of M1 NPs was increased to 10 μM (Figure 3A), indicating the excellent biocompatibility of M1 NPs. However, exposure to 808 nm laser irradiation at 1 W/cm^2^ for 10 min led to a significant decrease in cell viability with the increase of M1 NP concentration, and less than 5% cell viability remained at 10 μM concentration of M1 NPs, indicating the high phototoxicity of M1 NPs. Subsequently, the phototoxicity of M1 NPs to 4T1 cells was analyzed via a calcein-AM/propidium iodide (calcein-AM/PI) co-staining assay. As presented in Figure 3B, only the cells in the M1 NPs + laser group emitted red fluorescence, while the cells of the other three groups exhibited strong green fluorescence.

F-actin is an important component of the cytoskeleton, which is closely related to cell movement and growth.^30^ F-actin can be specifically stained by phalloidin to analyze the morphological changes by monitoring the green fluorescence of FITC-labeled phalloidin.^31^ F-actin was almost disrupted in 4T1 cells in the M1 NPs + laser group, while the three control groups showed a rigid filamentous structure (Figure 3C), indicating that the M1 NPs effectively damaged F-actin under laser irradiation, inhibiting the growth and proliferation of 4T1 cells.^32^ The flow cytometry results, provided in Figure 3D, also confirmed that M1 NPs could efficiently promote the apoptosis of irradiated 4T1 cells.^33^ The M1 NPs + laser group showed 42.67% early apoptosis and 55% late apoptosis, while the other three groups showed no significant apoptosis, implying that cell death was caused by M1 NP-induced apoptosis. Subsequently, the inhibitory effect of M1 NPs on DNA replication in 4T1 tumor cells was assessed by 5-ethynyl-2′-deoxyuridine (EdU) staining.^34,35^ As evident in Figure 3E, only the M1 NPs + laser group did not show green fluorescence, demonstrating the excellent inhibition of 4T1 cell proliferation by M1 NPs upon laser irradiation.

**Figure 4:**
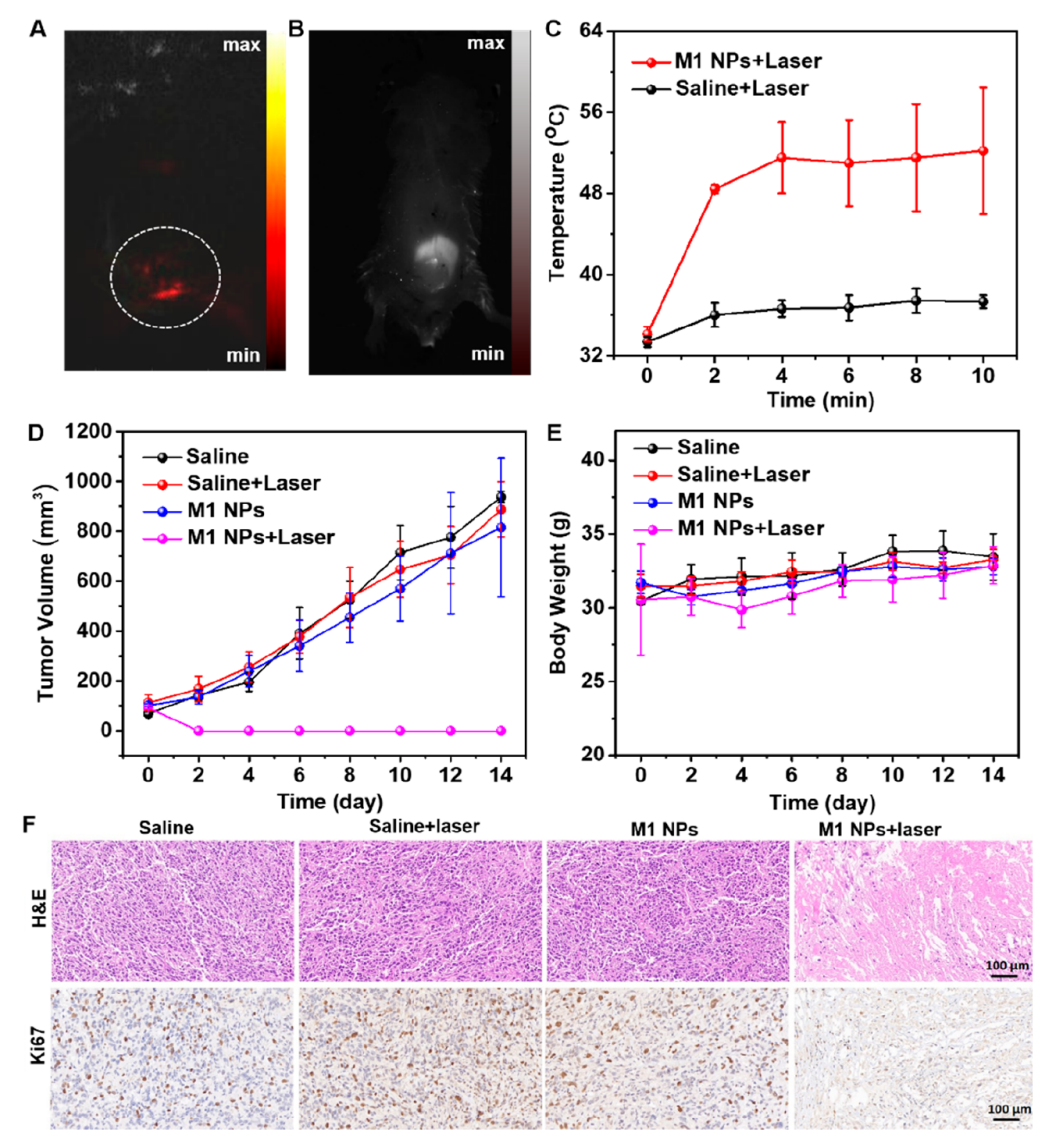
(A) PA imaging, (B) NIR-II fluorescence imaging, and (C) time-dependent temperature changes under 808 nm laser irradiation of tumor regions taken from 4T1 tumor-bearing mice (10 μM, 200 μL of M1 NPs or 200 μL of saline, 1 W/cm^2^). Change with time in tumor volumes (D) and body weights (E) of mice of different treatment groups. (F) H&E and Ki67-stained sections of tumors from different treatment groups.

High-resolution images are highly desirable for precise diagnosis and accurate guidance for tumor treatment. Taking the advantages of high PCE and NIR-II fluorescence emission of M1 NPs, the *in vivo* PA and NIR-II fluorescence imaging was performed.^36−39^ A strong PA and NIR-II fluorescence from the tumor regions are clearly observed in Figure 4A,B, respectively, after intratumoral injection of 4T1 tumor-bearing mice with M1 NP aqueous solution. Next, we evaluated the *in vivo* PTT efficacy of M1 NPs. When the tumor volume of 4T1 tumor-bearing mice reached 100 mm^3^, they were divided into four groups, viz. saline, saline + laser, M1 NPs, and M1 NPs + laser, and subjected to their respective treatments. The temperature of the tumor was recorded by infrared thermography. As illustrated in Figure 4C, the tumor temperature of the M1 NPs + laser group increased rapidly to 50 °C after 2 min, and afterward, it was maintained around 55 °C. However, the temperature of the saline + laser group increased only slightly, indicating that M1 NPs exhibit robust photothermal conversion ability *in vivo* as well. Over the next 14 days, tumor volumes and body weights of mice were monitored to assess the *in vivo* photothermal efficacy of M1 NPs. The tumors in the M1 NPs + laser group were completely ablated without recurrence during the follow-up, while the tumor volumes in the control groups continued to grow malignantly (Figure 4D). Moreover, the weight change of tumor-bearing mice in all groups remained consistent (Figure 4E), further demonstrating the good biocompatibility of M1 NPs. Next, tumors from different treatment groups were peeled and stained with hematoxylin&eosin (H&E), and Ki67 sections to assess the damage and proliferation of cancer cells.^40^ As shown in Figure 4F, only the tumor section from the M1 NPs + laser group exhibited a significant increase in the cytoplasmic gap, suggesting tumor cell damage and proliferation inhibition after PTT.

## 4 Conclusions

In summary, we selected a D-π-A structured hemicyanine dye M1 and obtained an NPs aqueous solution by encapsulating it into DSPE-PEG2000-NH_2_ (M1 NPs). We demonstrated that the aggregation could significantly enhance the photophysical performance of M1, including better photostability and higher photothermal conversion capability. The M1 NP aqueous solution exhibited strong NIR-I absorption and NIR-II fluorescence, making them excellent PA and NIR-II fluorescent imaging agents. The free rotation and the low *E*_g_ of M1 increased its PCE to 77.5%. M1 NPs were capable of inhibiting tumor cell DNA replication and backbone collapse through good photothermal properties, inducing apoptosis. Based on these excellent properties, the application of M1 NPs in PA and NIR-II fluorescence imaging-guided PTT of tumors was demonstrated. This work provides a benchmark for designing photothermal agents with NIR-II fluorescence and high PCE.

However, M1 NPs still have some disadvantages in the application of tumor diagnosis and treatment. For example, M1 NPs cannot generate ROS under laser irradiation, the incapability of achieving photothermal and photodynamic synergy to improve the therapeutic effect. The fluorescence wavelength and quantum yield could be increased for better fluorescence imaging. The tumor-targeting capability could be enhanced for targeting therapy. In general, the introduction of heavy atoms and paramagnetic groups, and increasing the torsion angles between electron donor and acceptor could improve the ROS generation capabilty.^41^ The introduction of aggregation-induced emission groups, such as triphenylamine, tetraphenylethylene, etc. can increase the fluorescence quantum yield and avoid fluorescence quenching.^42,43^ The surface modification of tumor-targeting probes, such as folic acid, bovine serum albumin, and tumor cell membrane could improve the tumor-targeting capability.^44−46^ While the couplling of quaternary ammonium or morpholine groups can improve the suborganelles targeting ability.^47−49^ These strategies may provide some thoughts for designing high efficient molecules for cancer treatment.

## Supporting Information Available

Dynamic light scattering particle size variation of M1 NPs; UV–vis absorption spectra of different concentrations of M1 in THF/H_2_O (v/v = 50/1950); thermal images of M1 NP aqueous solution; temperature increasing–decreasing curve of M1 NPs and pure water; ROS generating capability of M1 NPs; photostability of M1 NPs (PDF)
