# Supplementary material for: Aggregation-Enhanced Photophysical Performance of D-π-A Structured Hemicyanine for NIR-II Fluorescent and Photoacoustic Imaging-Guided Photothermal Therapy
Source: Chem Biomed Imaging. 2023 Mar 6;1(6):541–9. doi: 10.1021/cbmi.2c00004 (PMC11503799; doi:10.1021/cbmi.2c00004)
Supplement: Supplementary file 1 — im2c00004_si_001.pdf [file im2c00004_si_001.pdf]

Supporting Information

Aggregation Enhanced Photophysical Performance of D-π-A Structured Hemicyanine for NIR-II Fluorescent and Photoacoustic Imaging-guided Photothermal Therapy

Baoling Li,[‡,a] *E Pang,*[‡,a] *Shaojing Zhao,*[a] *Guowei Deng,*[*,b] *Shuodong Wang,*[c] *Benhua Wang,*[a] *Jieyun Wu,*[d] *Guangle Niu,*[e] *Xiangzhi Song,*[a] *and Minhuan Lan*[*,a]

[a] Hunan Provincial Key Laboratory of Micro & Nano Materials Interface Science, College of Chemistry and Chemical Engineering, Central South University, Changsha, Hunan, 410083, P. R. China. Email: minhuanlan@csu.edu.cn

[b] College of Chemistry and Life Science, Sichuan Provincial Key Laboratory for Structural Optimization and Application of Functional Molecules, Chengdu Normal University, Chengdu, Sichuan, 611130, P.R. China. Email: Guoweideng86@163.com

[c] State Key Laboratory of Chemo/Biosensing and Chemometrics, College of Chemistry and Chemical Engineering, Hunan University, Changsha, Hunan, 410082, P. R. China.

[d] School of Optoelectronic Science and Engineering, University of Electronic Science and Technology of China, Chengdu, Sichuan, 611731, P.R. China.

[e] State Key Laboratory of Crystal Materials, Shandong University, Jinan, Shandong, 250100, P. R. China.

‡ These authors contributed equally to this work.

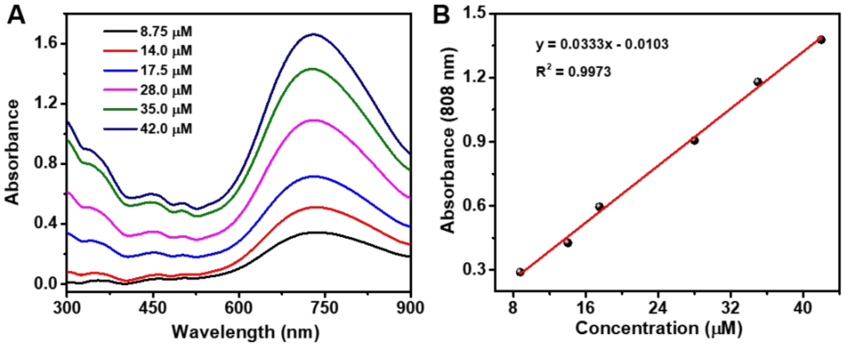

Figure S1 (A) UV-vis absorption spectra and (B) linear relationship plots of different concentrations of M1 in THF/H_2O(v/v=50/1950).

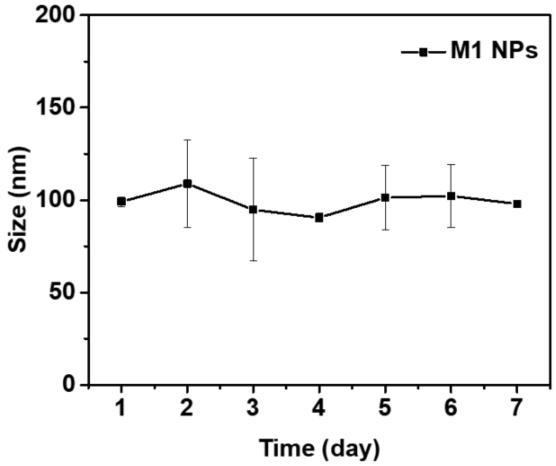

Figure S2 Dynamic light scattering particle size variation of M1 NPs at 7 days.

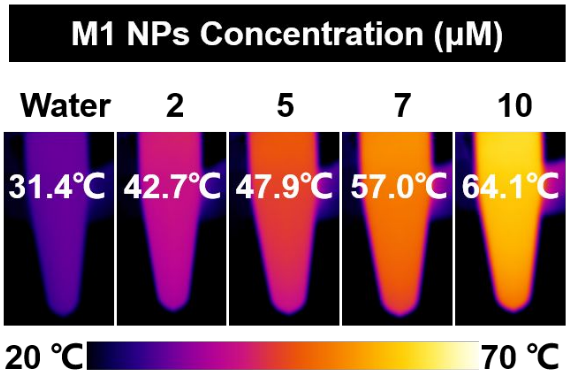

Figure S3 Thermal images of M1 NPs aqueous solution with different concentrations exposed laser irradiation.

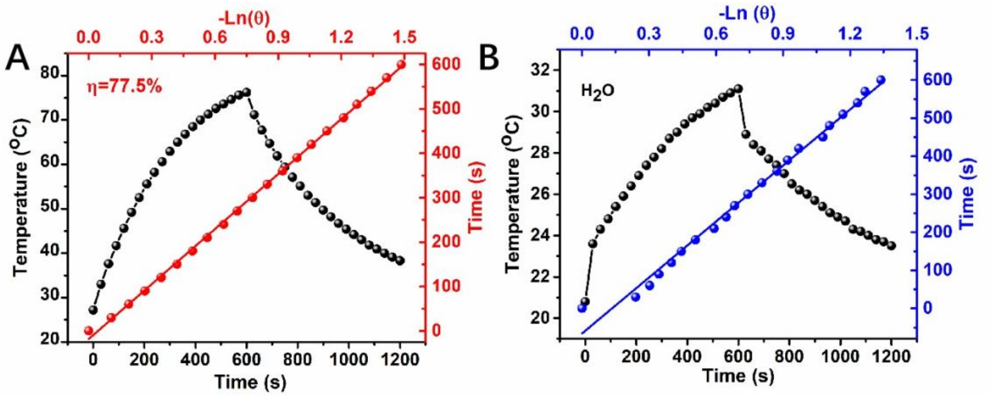

Figure S4 Temperature increasing-decreasing curve (black dot line) and plot of cooling time vs -Ln (θ) (red dot line) of (A) M1 NPs and (B) H_2O.

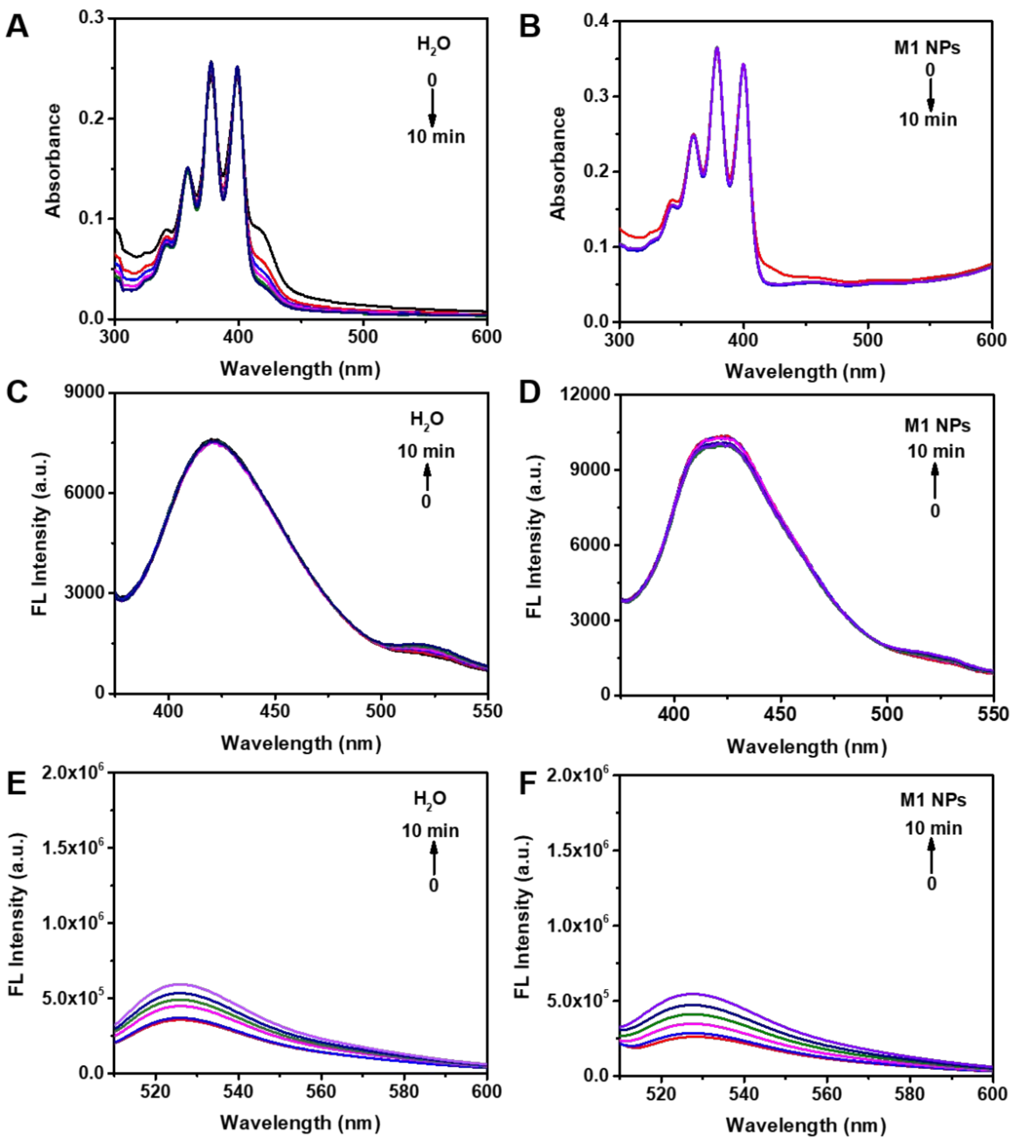

Figure S5 Laser irradiation time-dependent UV-vis absorption spectra of ABDA sodium salt aqueous solutions in the (A) absence and (B) presence of M1 NPs. Laser irradiation time-dependent fluorescence spectra of (C, D) TA sodium salt (λ_{ex}=320 nm) and (E, F) DHR123 (λ_{ex}=500 nm) aqueous solutions in the (C, E) absence and (D, F) presence of M1 NPs. Laser: 808 nm, 1.0 W/cm^2.

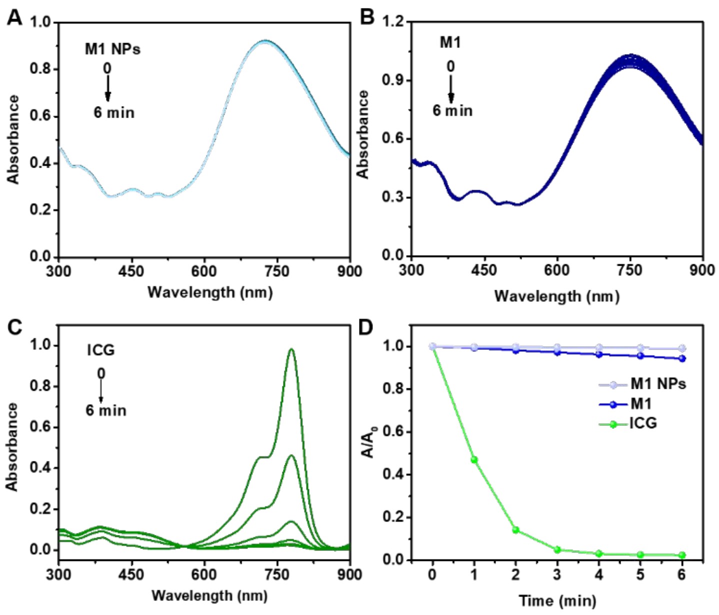

Figure S6 Time-dependent absorption spectra of (A) M1 (in DMSO), (B) M1 NPs and (C) ICG aqueous solutions under 808 nm laser irradiation (1 W/cm^2, 6 min); (D) The normalized absorbance variation of M1 (in DMSO), M1 NPs, and ICG after laser irradiation.
